# Gastric adenocarcinoma appearance in leiomyoma: A case report

**DOI:** 10.1016/j.ijscr.2020.05.050

**Published:** 2020-05-29

**Authors:** Kaoru Wada, Hirofumi Tazawa, Toshiaki Komo, Naoto Hadano, Takashi Onoe, Takeshi Sudo, Yosuke Shimizu, Kazuya Kuraoka, Takahisa Suzuki, Hirotaka Tashiro

**Affiliations:** a Department of Internal Medicine, National Hospital Organization, Kure Medical Center, Chugoku Cancer Center, 3-1 Aoyama, Kure City, Hiroshima, 737-0023, Japan; b Department of Surgery, National Hospital Organization, Kure Medical Center, Chugoku Cancer Center, 3-1 Aoyama, Kure City, Hiroshima, 737-0023, Japan; c Department of Diagnostic Pathology, National Hospital Organization, Kure Medical Center, Chugoku Cancer Center, 3-1 Aoyama, Kure City, Hiroshima, 737-0023, Japan; d Department of Gastroenterological and Transplant Surgery, Applied Life Sciences, Institute of Biomedical and Health Sciences, Hiroshima University, 1-2-3, Kasumi, Minami-ku, Hiroshima 734-8551, Japan

**Keywords:** SMT, submucosal tumor, EGD, esophagogastroduodenoscopy, EUS, endoscopic ultrasonography, CEA, carcinoembryonic antigen, CA 19-9, cancer antigen 19-9, CT, computed tomography, sHGG, solitary heterotopic gastric gland, GIST, gastrointestinal stromal tumor, Solitary heterotopic gastric gland, Adenocarcinoma in leiomyoma

## Abstract

**Introduction:**

We experienced an extremely rare case of gastric adenocarcinoma wrapped by leiomyoma.

**Presentation of case:**

A 65-year-old man had an abnormality (filling defect) of the upper gastrointestinal series in his first medical checkup five years prior. Esophagogastroduodenoscopy detected a 10 mm submucosal tumor-like lesion in the greater curvature of the upper gastric remnant body. Despite repeated biopsy from the lesion, there was no sign of malignancy. A delle was observed on the top of the tumor at another visit five year after the first and a biopsy specimen revealed poorly differentiated adenocarcinoma. Therefore, laparoscopic gastrectomy was performed. Histological assessment revealed a 28 × 22 mm elevated lesion with a slight depression. Microscopically, papillary adenocarcinoma was observed at the submucosa with a solitary heterotopic gastric gland adjacent to the lesion. The final diagnosis was papillary adenocarcinoma arising from a solitary heterotopic gastric gland in the leiomyoma. No recurrence has occurred during a follow-up of two and a half years after surgery.

**Conclusions:**

This is the first report of gastric adenocarcinoma arising from a submucosal tumor.

## Highlights

• Gastric adenocarcinoma arising from a leiomyoma which is diagnosed by pathological evidence as heterotopic glands carcinoma within it.
• Pathological evidences diagnosed heterotopic gastric glands within the leiomyoma, then adenocarcinoma arose from the heterotopic gastric glands.
• This is the first report that describes a case of gastric adenocarcinoma arising from leiomyoma.

## 1 Introduction

We experienced an extremely rare case of gastric adenocarcinoma wrapped by leiomyoma. Gastric leiomyoma is a submucosal growth that accounts for 2.5 % of gastric tumors. Some cases are clinically evident because of bleeding from ulceration of the overlying gastric mucosa [1]. This is the first report of adenocarcinoma arising from a submucosal tumor (SMT). This case report has been prepared in line with the SCARE criteria [2].

## 2 Presentation of case

**Fig. 1:**
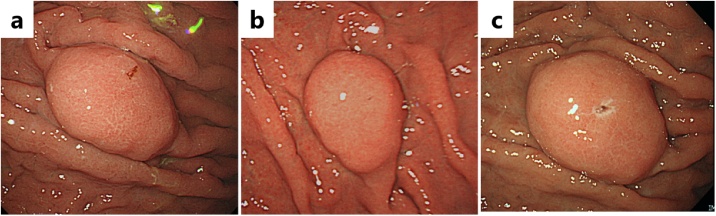
**a.** Esophagogastroduodenoscopy revealed a 10 mm submucosal tumor-like lesion in the greater curvature of the upper gastric remnant body five years prior. b. The tumor size grew to 14 mm in the three years prior. c. The tumor size had grown to 16 mm when performing biopsy with no malignancy one year prior. (short arrows) and internal liquid shown (long arrows).

**Fig. 2:**
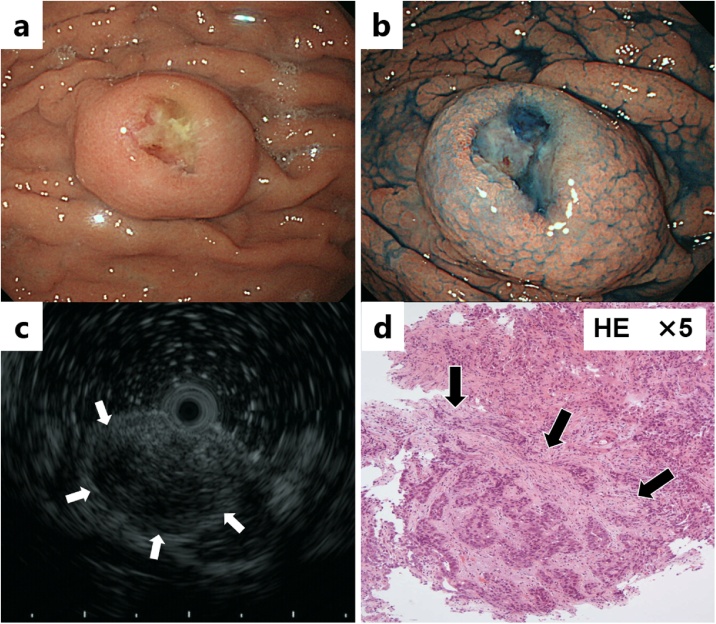
**a, b.** Esophagogastroduodenoscopy revealed a delle on the top of the tumor. **c.** Endoscopic ultrasonography revealed a hypo-echoic tumor located in the third layer (white arrows). **d.** Biopsy specimen revealed poorly differentiated adenocarcinoma.

**Fig. 3:**
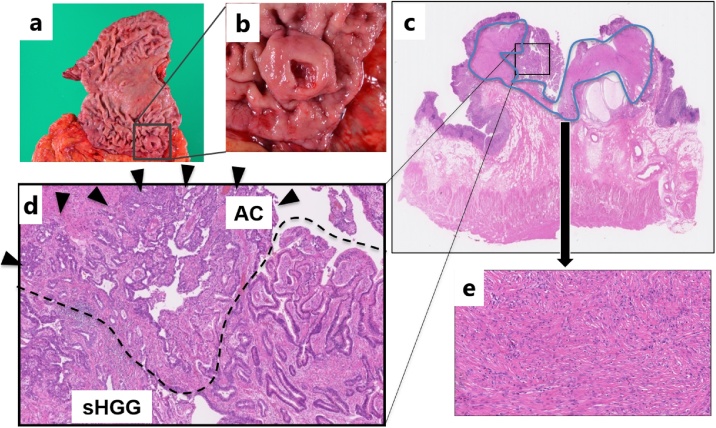
**a.** The picture shows a resected specimen. The resected tumor was 25 mm in size surrounded by black square. **b.** Histological assessment revealed a 28 × 22 mm ulcerative protrusion. Adenocarcinoma was detected in part of yellow line. **c, d, e.** Microscopically, papillary adenocarcinoma (AC: arrow heads) was observed at the submucosa with solitary heterotopic gastric gland (sHGG: dotted line) adjacent to the lesion (hematoxylin-eosin staining, original magnification: × 400). There are smooth muscle tissues (surrounded by the blue line) around the adenocarcinoma and sHGG.

**Fig. 4:**
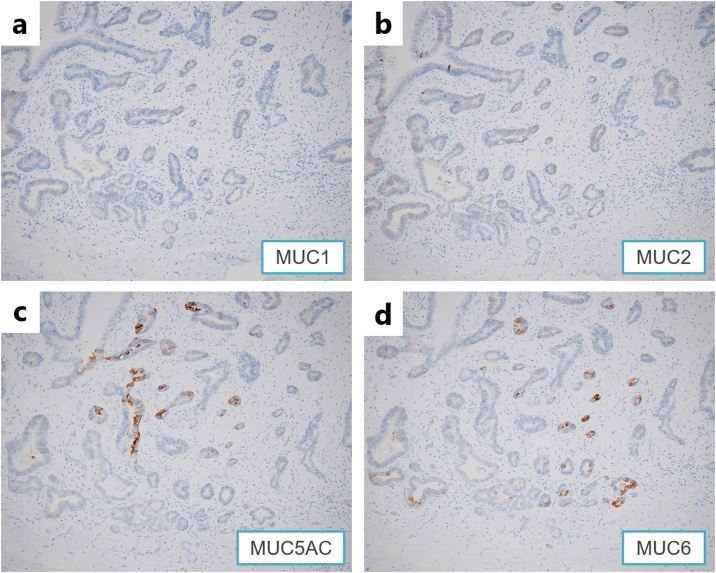
Immunohistochemical stainings are shown below. **a.** MUC1 was negative. **b.** MUC2 was negative. **c.** MUC5AC was positive. **d.** MUC6 was positive.

A 65-year-old man had an abnormality (filling defect) of the upper gastrointestinal series in his first medical checkup five years prior. The patient had undergone distal gastrectomy for a gastric ulcer at age forty. Esophagogastroduodenoscopy (EGD) revealed a 10 mm submucosal tumor-like lesion in the greater curvature of the upper gastric remnant body. Endoscopic ultrasonography (EUS) revealed a hypo-echoic tumor located in the third layer. Blood test findings revealed a carcinoembryonic antigen level of 3.8 ng/mL and carbohydrate antigen 19−9 level of 6 U/mL, indicating the tumor markers were within the normal range. Because there was no sign of malignancy, the gastric lesion was followed up by annual EGD (Fig. 1). A delle was observed on the top of the tumor five year after the first visit and a biopsy specimen revealed poorly differentiated adenocarcinoma (Fig. 2). Contrast computed tomography (CT) showed no nodal or distant metastasis. Laparoscopic gastrectomy and lymph node dissection for remnant gastric cancer was performed. Histological assessment revealed a 28 × 22 mm elevated lesion with a slight depression. Microscopically, papillary adenocarcinoma was observed with a solitary heterotopic gastric gland (sHGG) surrounding by smooth muscle tissues (Fig. 3). Immunohistochemical staining of sHGG, MUC5AC and MUC6 were positive, and MUC1 and MUC2 were negative (Fig. 4). These results revealed the sHGG originated from the stomach. There was smooth muscle tissue around the adenocarcinoma and sHGG was found only in the submucosa. Immunohistochemical staining of the smooth muscle tissues revealed SMA and Vimentin/desmin were positive, S-100a was weak positive, and C-kit/CD34 was negative (data not shown). The final diagnosis was papillary adenocarcinoma arising from a solitary heterotopic gastric gland in the leiomyoma [pT2(MP)N0M0, ly0(D2-40), v0, pPM0, pDM0: according to the Japanese classification of gastric carcinoma]. There has been no recurrence during follow-up of two and a half years after surgery.

## 3 Discussion

We experienced an extremely rare case of gastric adenocarcinoma wrapped by leiomyoma. However, it is impossible to explain that the adenocarcinoma arose from the leiomyoma. The SMT had been observed for 5 years. The tumor appearance including its mucosal surface had never changed for 4 years, and a delle appeared on top of the tumor. The pathological findings showed that carcinoma was not present at the surface mucosa, only at fundus of the ulcer. This finding indicated that the adenocarcinoma had arisen from the SMT. There is no previous report of adenocarcinoma arising from SMT.

There are many reports about the adenocarcinoma from heterotopic gastric glands [[3], [4], [5]]. The HGG is considered benign and is associated with malignant transformation [6]. The transforamtion is generally explained by HGG arising from gastric glands that exist congenitally in the submucosa, or from aberration of the epithelium into the submucosa as a result of repeated erosion and regeneration of the mucosa [7,8]. It has been reported that both HGG and gastric cancer develop as a result of repeated erosion and regeneration of the mucosa, suggesting that submucosal HGG are paracancerous lesions [9,10]. There are no previous reports of an association between submucosal HGG and leiomyoma, however one report presents a case of a gastrointestinal stromal tumor (GIST) with an unusual glandular component with typical low-grade spindle cell patterns of GIST intermingled with numerous and partly cystic glands [11].

In summary, we first diagnosed heterotopic gastric glands within the leiomyoma, then adenocarcinoma arose from the heterotopic gastric glands. Supporting pathological evidence shows that there is no connection between adenocarcinoma and the normal mucosa lamina propria, and there is no finding of inversion of the mucous membrane. This is the first report of gastric adenocarcinoma appearance in leiomyoma.

HGG is classified into 4 types based on their number and range of distribution: solitary type with 3 sites or less, localized type with 4–9 sites at the focal area, broad type with 4–9 sites in the broad area, and diffuse type with at least 10 sites which exist in the entire stomach [12]. The diffuse type is seen in 98 % of gastric cancer complications, and the rate of complication with multiple gastric cancers is reported as 32 % [13]. Currently, in the Japanese classification of gastric carcinoma, there is no definition concerning the depth of tumor invasion that spreads to HGG in the submucosa [14]. It may be better to choose total gastrectomy for gastric cancer arising from the diffuse type HGG. In the present case, HGG was the localized type because the area was just inside of the SMT. We performed remnant gastrectomy. No recurrence has occurred during a follow-up of two and a half years after surgery.

## 4 Conclusions

This is the first report that describes a case of gastric adenocarcinoma arising from leiomyoma.

## Declaration of Competing Interest

None of the authors has anything to disclose.

## Funding

None of the authors has anything to disclose.

## Ethical approval

All procedures used in this research were approved by the Ethical Committee of National Hospital Organization, Kure Medical Center, Chugoku Cancer Center.

## Consent

Written informed consent was obtained from the patient for the publication of this case report and any accompanying images. A copy of the written consent form is available for review by the Editor-in-Chief of this journal.

## Author contribution

Hirofumi Tazawa is the corresponding author and carried out revision of the manuscript. Takashi Suzuki performed the surgery. Toshiaki Komo participated in the surgery. Kaoru Wada participated in the clinical treatments. Kazuya Kuraoka performed the pathological analysis. Hirotaka Tashiro and Takahisa Suzuki supervised the writing of the manuscript. All authors read and approved the final manuscript.

## Registration of research studies

This is not a research report, just a case report. So we do not need register this.

## Guarantor

Hirofumi Tazawa has accepted full responsibility for this work and the decision to publish it.

## Provenance and peer review

Not commissioned, externally peer-reviewed.
